# Supplementary material for: Development of chemokine network inhibitors using combinatorial saturation mutagenesis
Source: Commun Biol. 2025 Apr 3;8:549. doi: 10.1038/s42003-025-07778-6 (PMC11969024; doi:10.1038/s42003-025-07778-6)
Supplement: Supplementary file 4 — Supplementary Data 2 [file 42003_2025_7778_MOESM4_ESM.zip › SupplementaryData3/scripts/xattune_ci.tar!/./script/analysis/JK/JK_CMA_ExactPvalues.docx]

Supplementary Table: Exact P values for cell migration experiments, comparisons
to control

| ID | Experiment components | Control | Number of biological replicates | P value |
| --- | --- | --- | --- | --- |
| Combinatorial | HD2Y5W | HD2Y5W | 40 | NA |
| Combinatorial | HD2 | HD2Y5W | 40 | 3.40e-01 |
| Combinatorial | HD2SCR | HD2Y5W | 40 | 3.84e-06 |
| Combinatorial | HD2T6D | HD2Y5W | 31 | 1.00e+00 |
| Combinatorial | HD2T6W | HD2Y5W | 31 | 1.00e+00 |
| Combinatorial | HD2A9W | HD2Y5W | 13 | 6.66e-01 |
| Combinatorial | HD2L11I | HD2Y5W | 31 | 1.85e-01 |
| Combinatorial | HD2T12W | HD2Y5W | 31 | 7.67e-01 |
| Combinatorial | HD2T16C | HD2Y5W | 28 | 1.27e-01 |
| Combinatorial | HD2CM304 | HD2Y5W | 31 | 8.33e-01 |
| Combinatorial | HD2CM307 | HD2Y5W | 31 | 1.10e-02 |
| Combinatorial | HD2CM452 | HD2Y5W | 31 | 1.00e+00 |
| Combinatorial | HD2CM526 | HD2Y5W | 25 | 8.23e-01 |
| Combinatorial | HD2CM539 | HD2Y5W | 28 | 2.86e-01 |
